# Supplementary material for: Holliday junction–ZMM protein feedback enables meiotic crossover assurance
Source: Nature. 2025 Sep 24;647(8090):766–75. doi: 10.1038/s41586-025-09559-x (PMC12630000; doi:10.1038/s41586-025-09559-x)

nature portfolio

https://doi.org/10.1038/s41586-025-09559-x

Supplementary information

Holliday junction–ZMM protein feedback enables meiotic crossover assurance

In the format provided by the authors and unedited

Supplementary Figure 1: Gel source data

Uncropped gel images. Cropped areas are indicated by dashed boxes.
Western blots are from the same gel unless otherwise noted.

Figure 1i and Extended Data Figure 3c

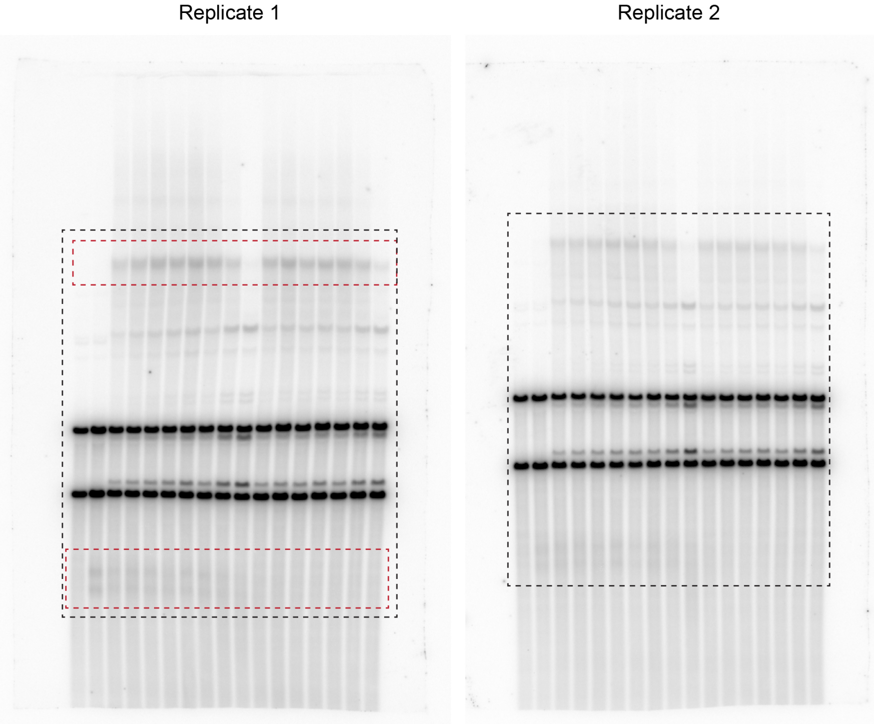

Figure 2d

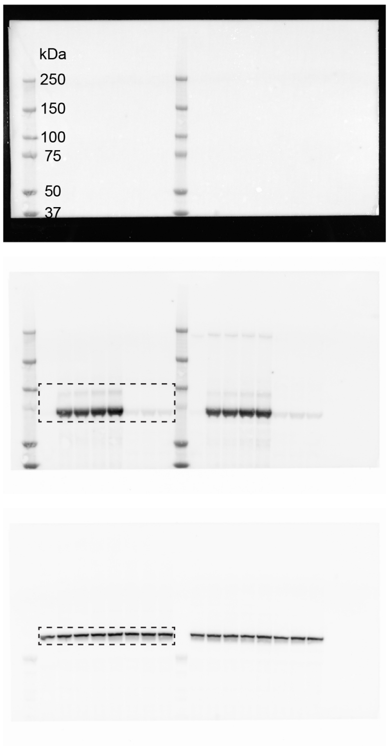

Figure 2j

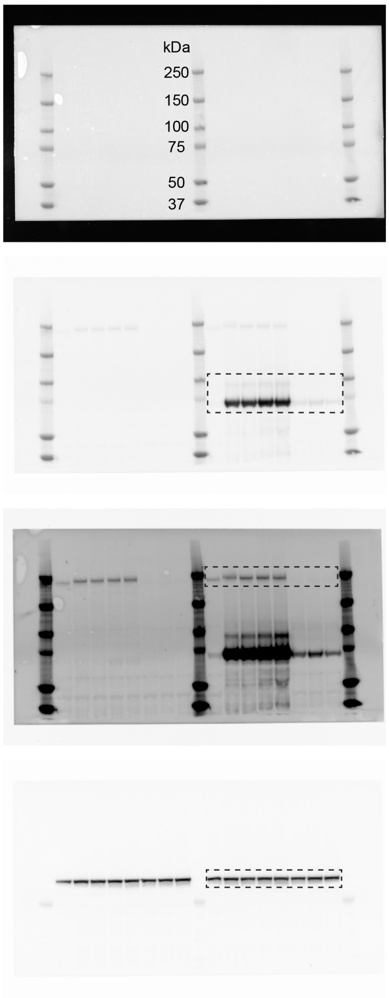

Figure 2g and k

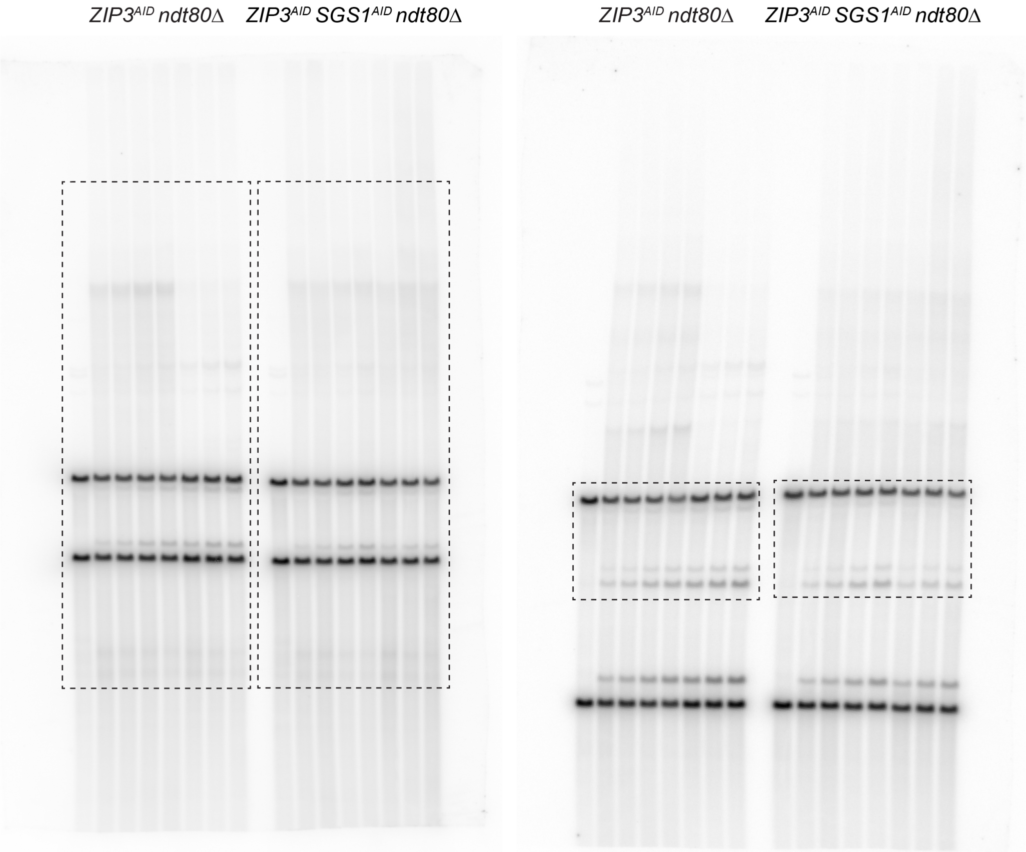

Supplementary Figure 1: Gel source data (continued)

Figure 3d and Extended Data Figure 6e

Replicate 1 Replicate 2

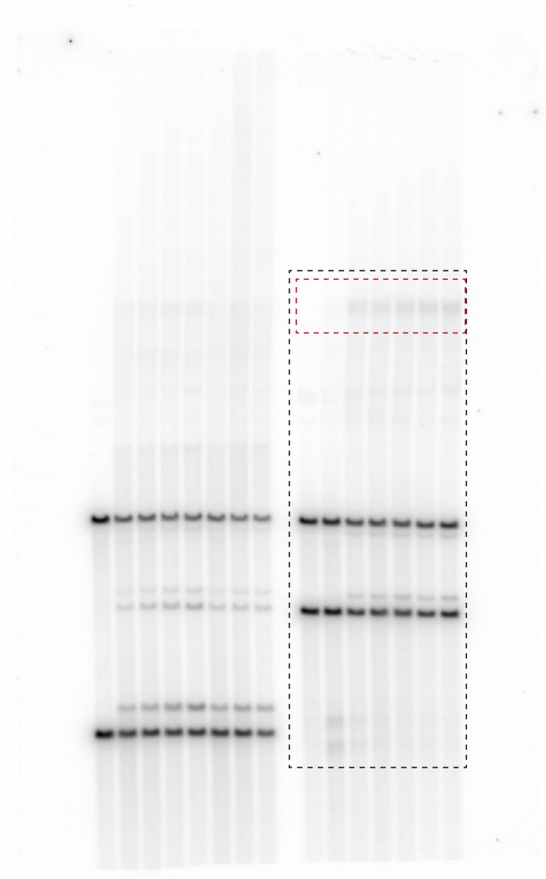

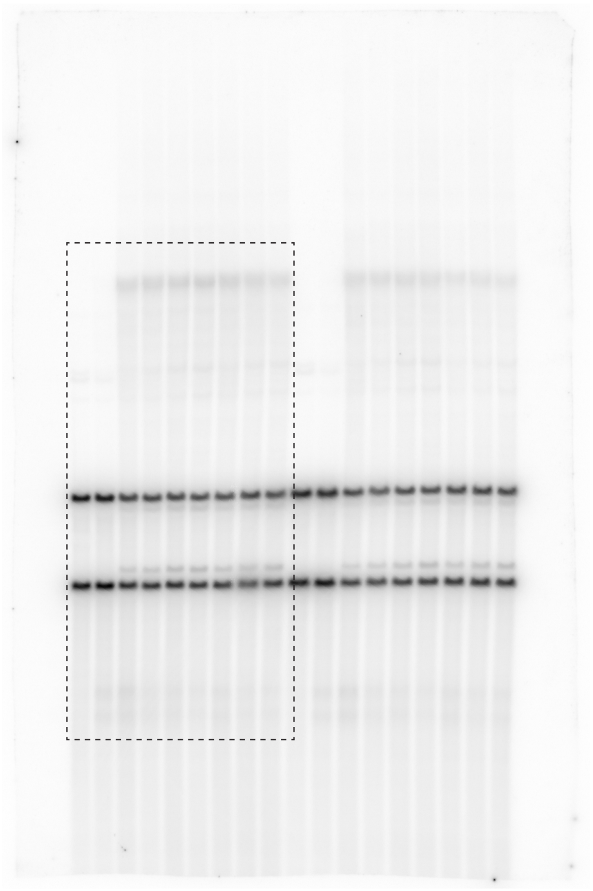

Figure 4c

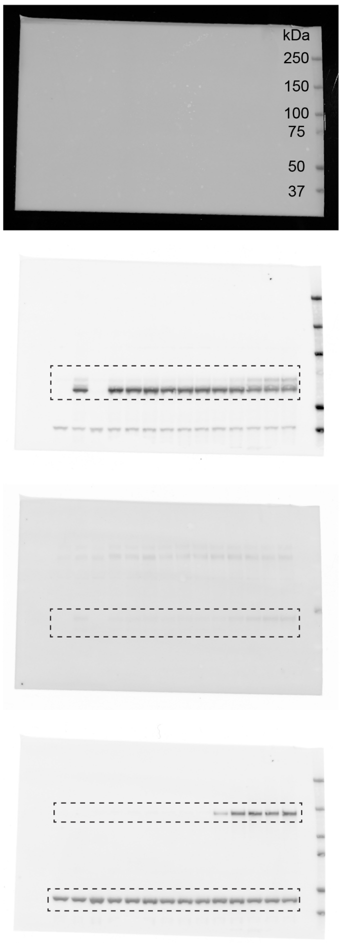

Figure 4d and Extended Data Figure 8c

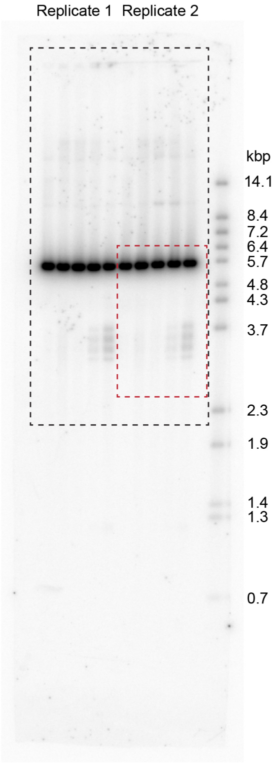

Figure 4f

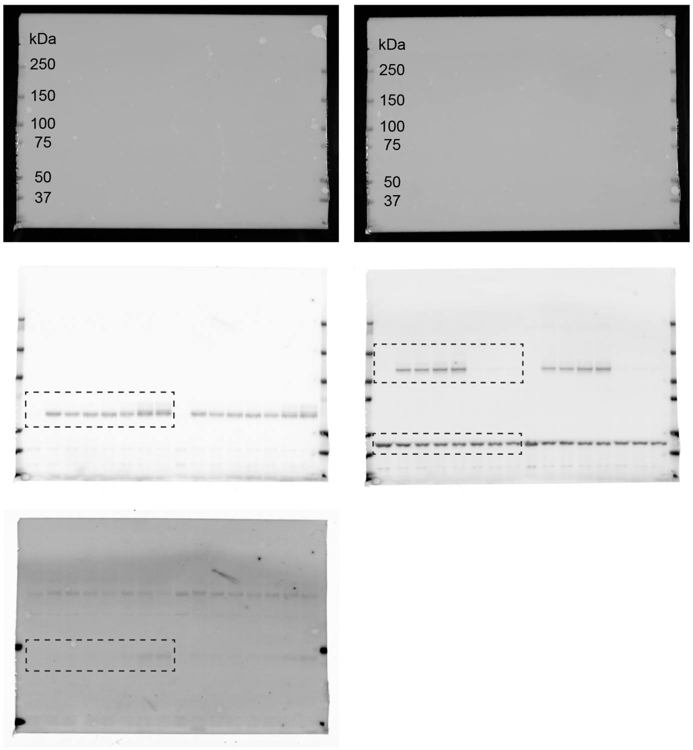

Supplementary Figure 1: Gel source data (continued)

Figure 4g and Extended Data Figure 8e

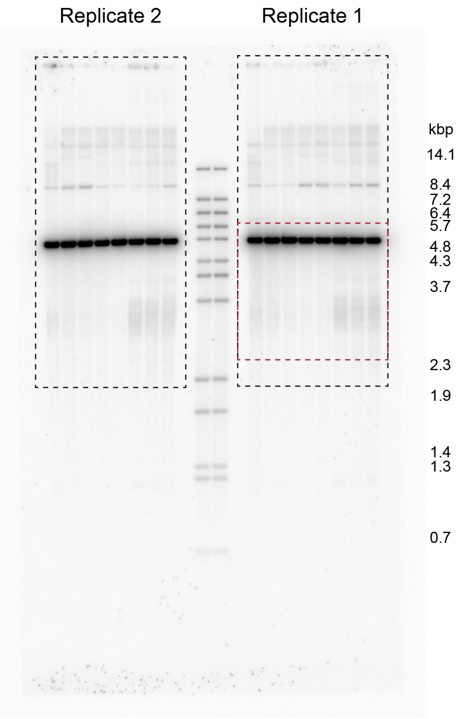

Extended Data Figure 1b

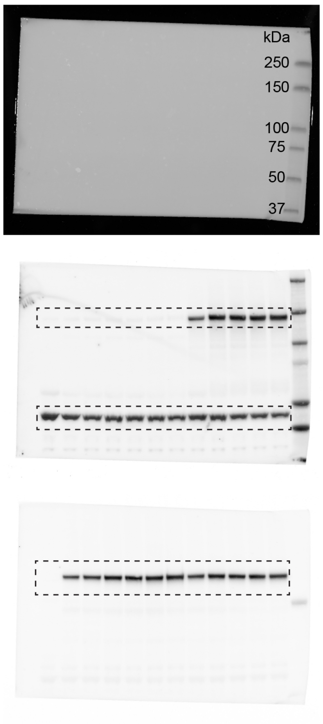

Extended Data Figure 1d

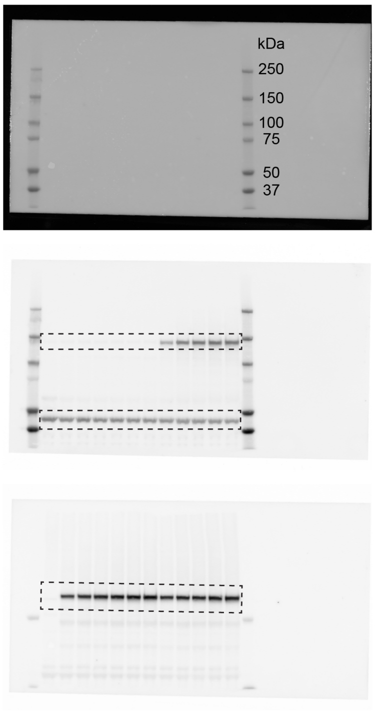

Extended Data Figure 3d

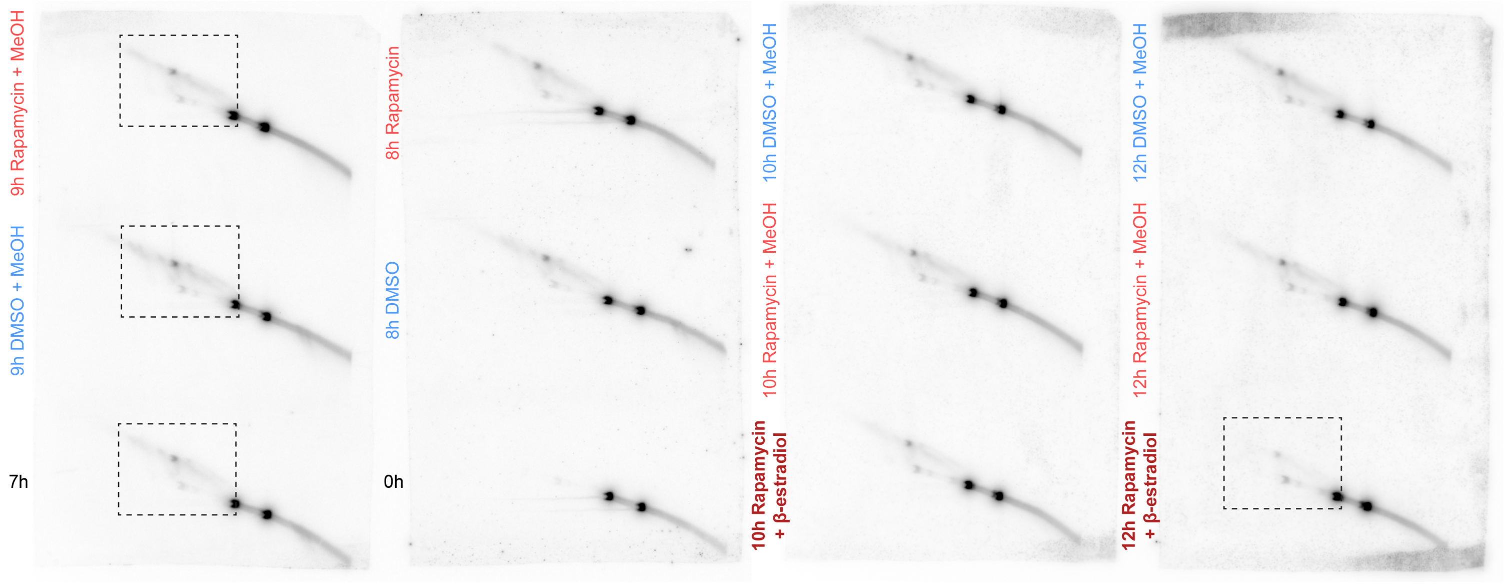

Extended Data Figure 3f

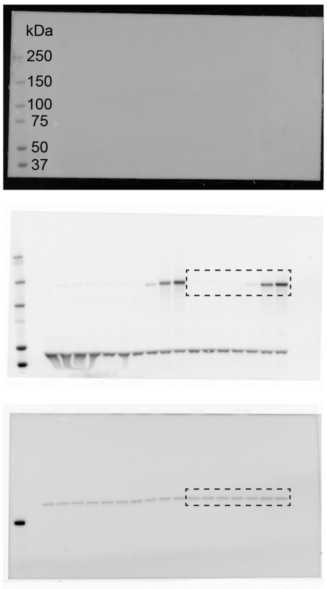

Extended Data Figure 4f

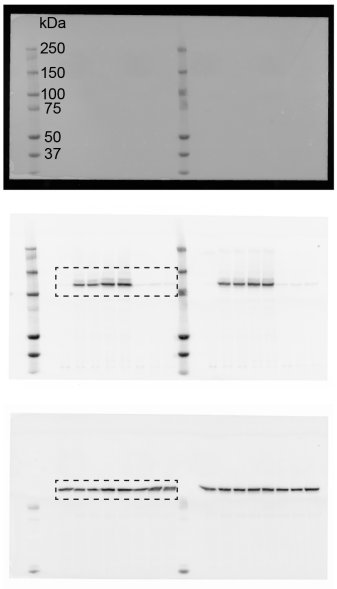

Extended Data Figure 4g

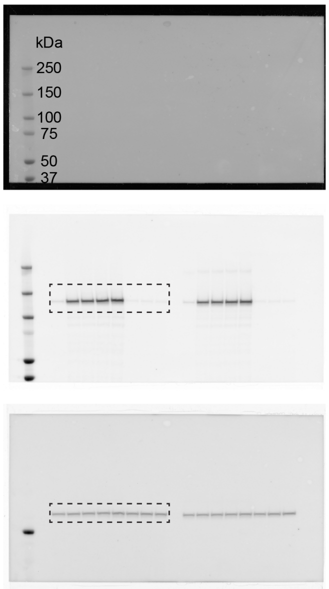

Supplementary Figure 1: Gel source data (continued)

Extended Data Figure 5a and d

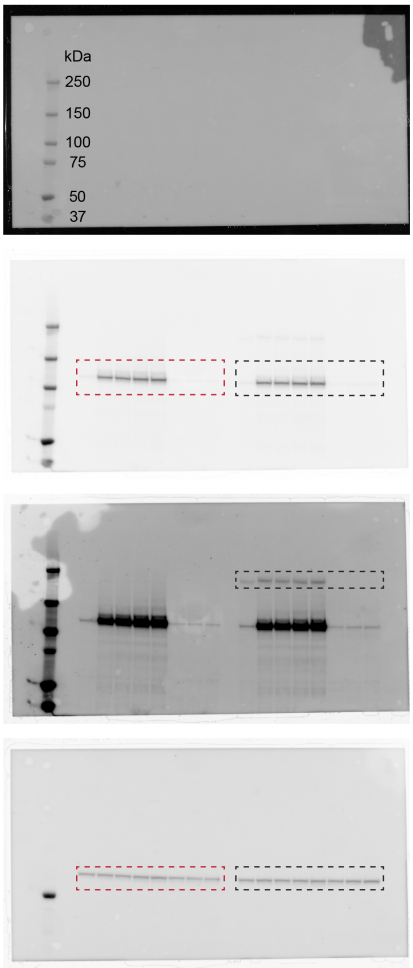

Extended Data Figure 5b

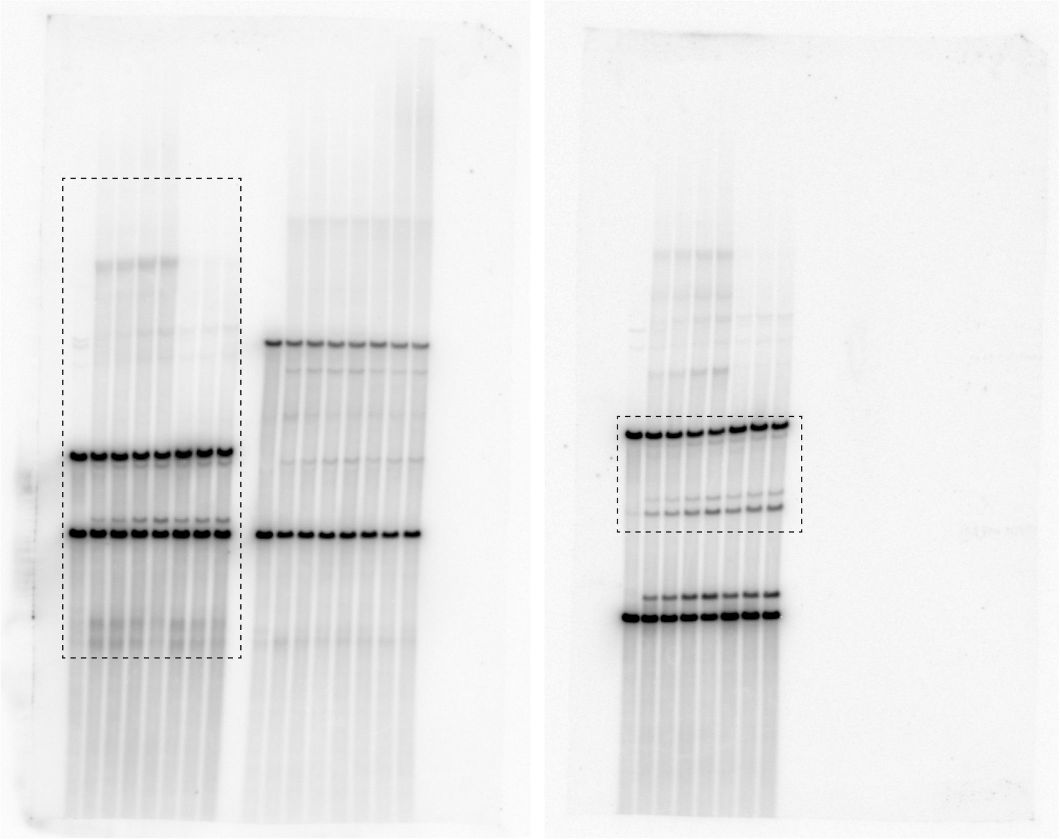

Extended Data Figure 5e

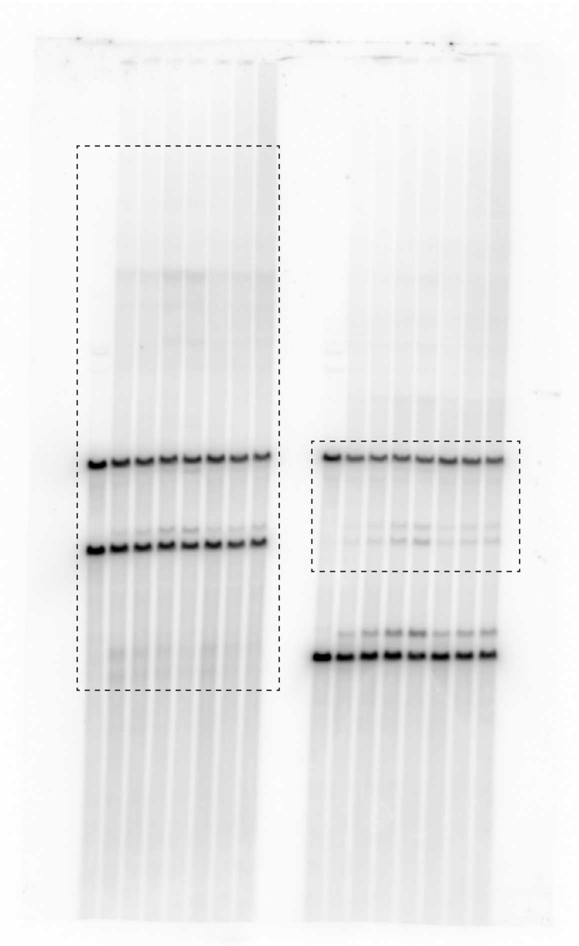

Extended Data Figure 5g

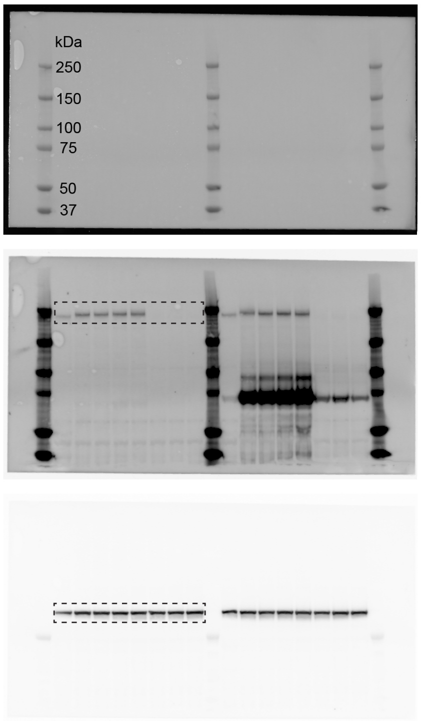

Supplementary Figure 1: Gel source data (continued)

Extended Data Figure 5h

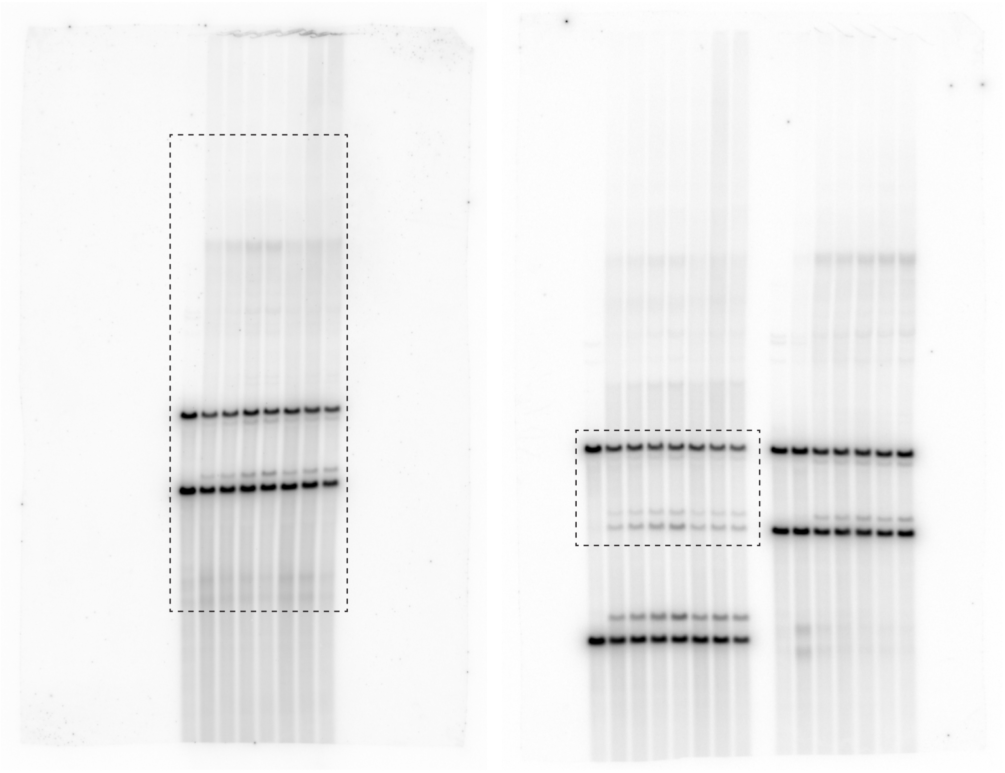

Extended Data Figure 5o

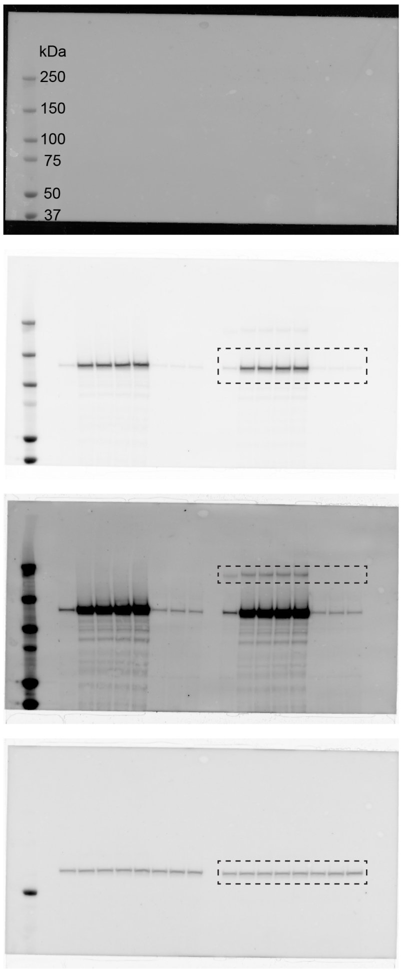

Extended Data Figure 6c

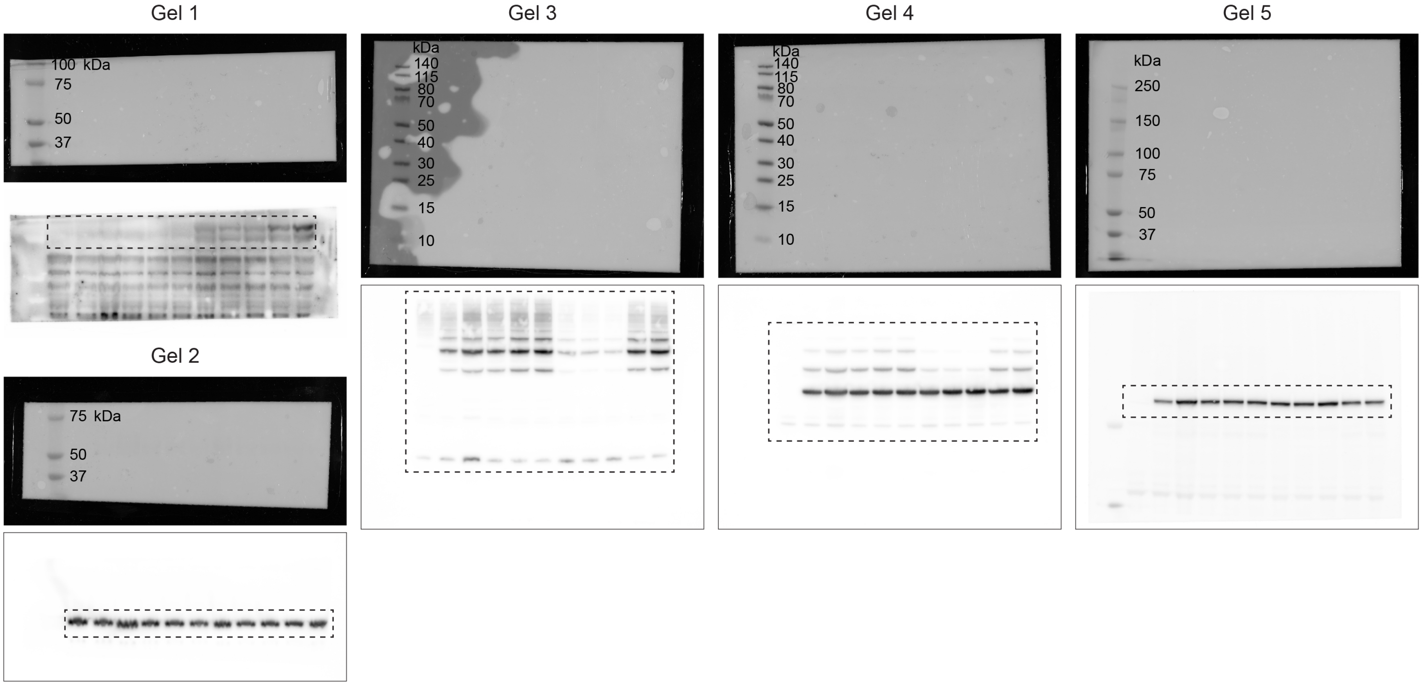

Supplementary Figure 1: Gel source data (continued)

Extended Data Figure 6i

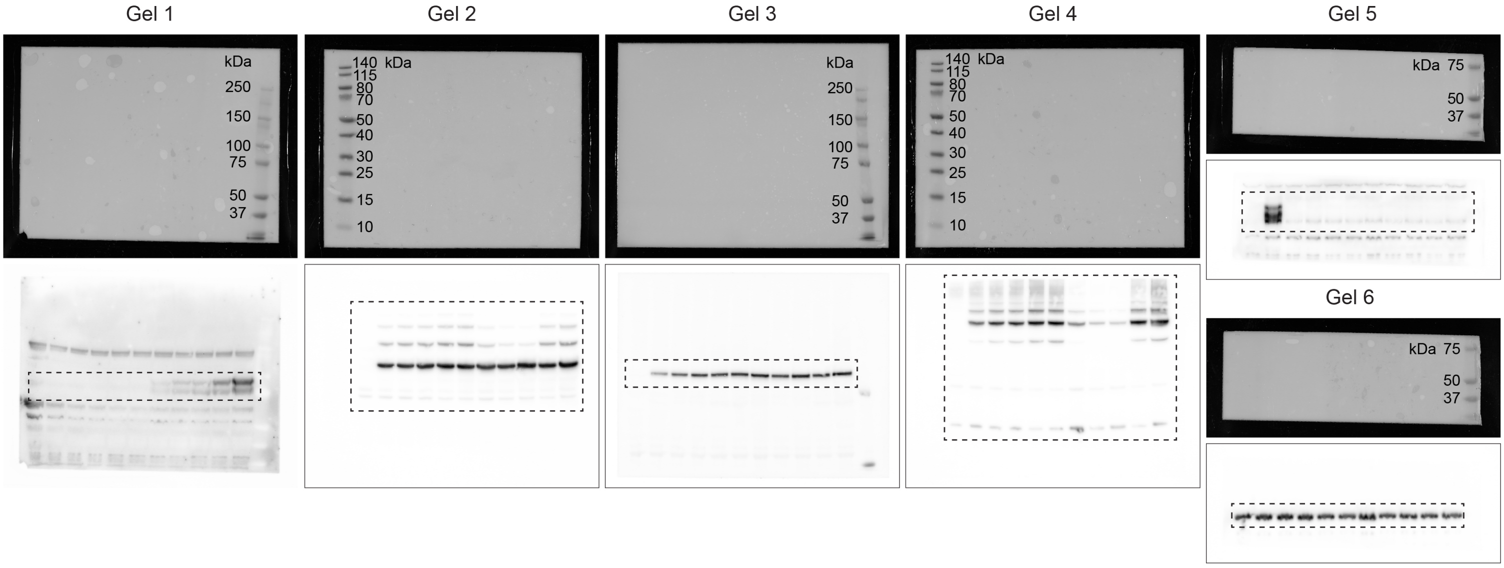

Extended Data Figure 7a

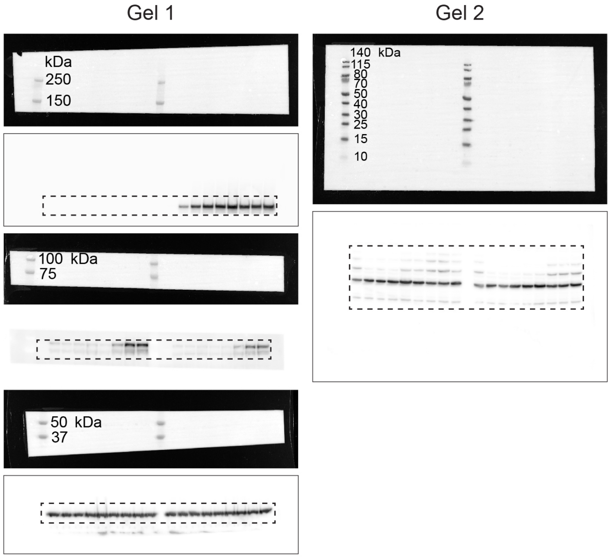

Extended Data Figure 8b

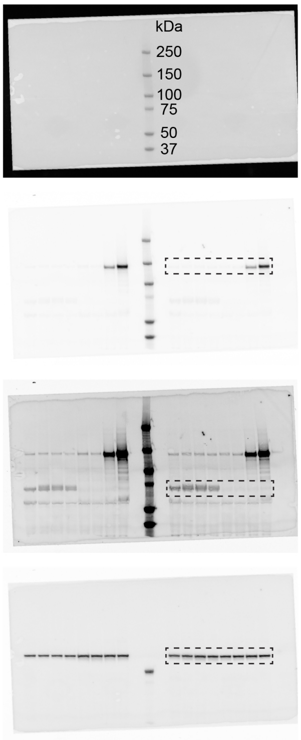

Extended Data Figure 8d

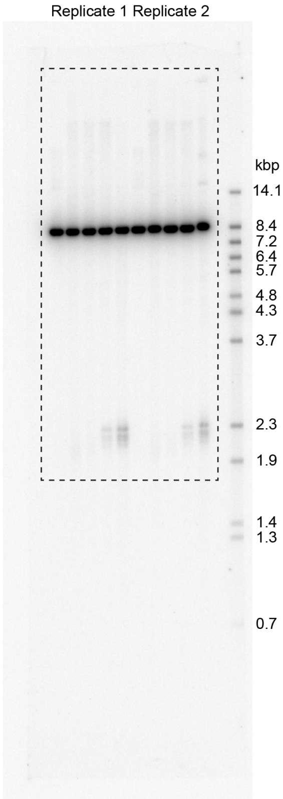

Extended Data Figure 8f

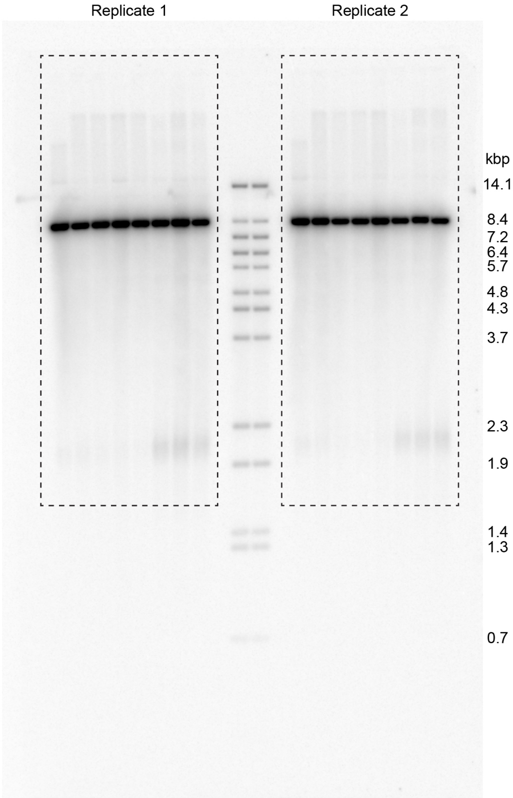

Supplementary Figure 1: Gel source data (continued)

Extended Data Figure 8g

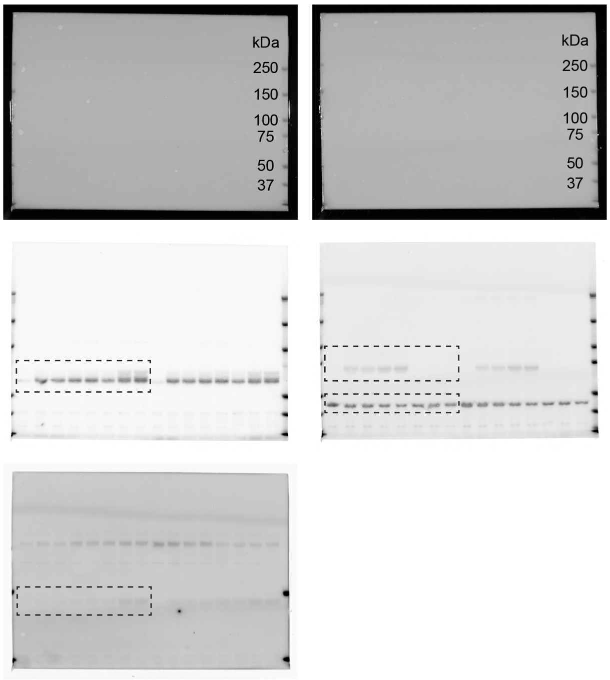

Extended Data Figure 8h

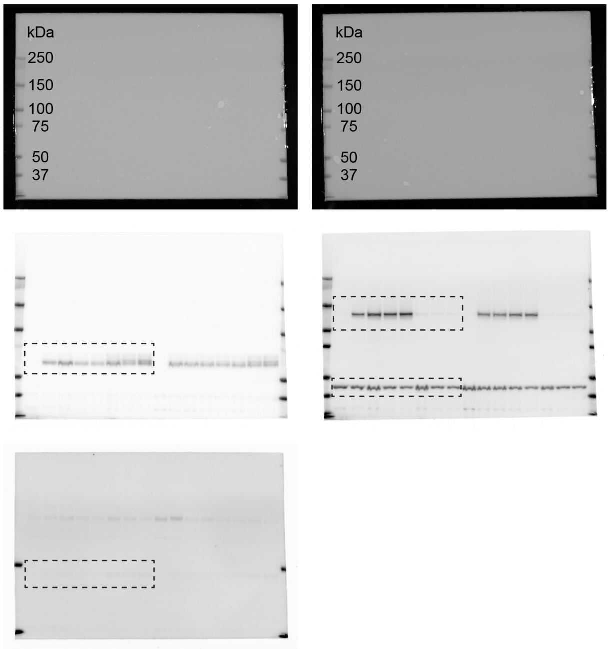

Extended Data Figure 9c

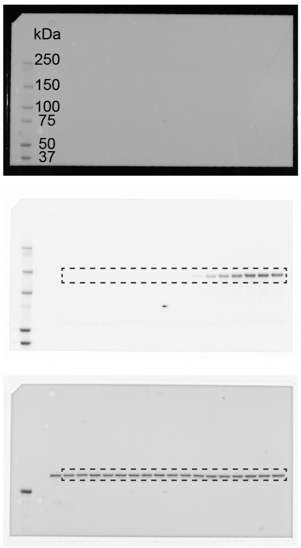

Extended Data Figure 10b

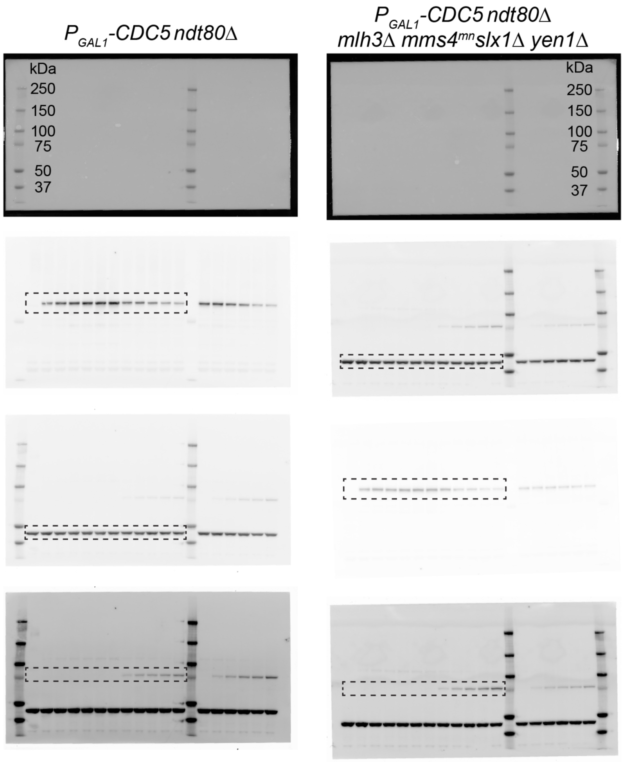

Extended Data Figure 10f

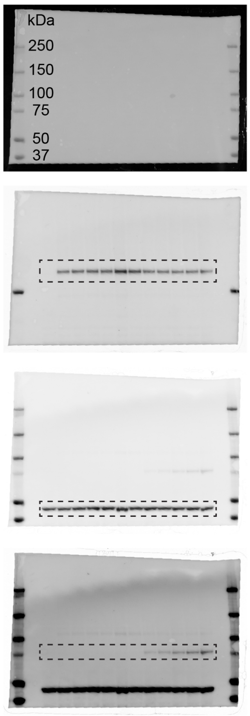

Supplement: Supplementary file 1 — The uncropped Southern and western blot images generated in this study. [file 41586_2025_9559_MOESM1_ESM.pdf]
