# Supplementary material for: Holliday junction–ZMM protein feedback enables meiotic crossover assurance
Source: Nature. 2025 Sep 24;647(8090):766–75. doi: 10.1038/s41586-025-09559-x (PMC12630000; doi:10.1038/s41586-025-09559-x)
Supplement: Supplementary file 2 — Reporting Summary [file 41586_2025_9559_MOESM2_ESM.pdf]

nature portfolio

Corresponding author(s): Joao Matos
Last updated by author(s): Jul 15, 2025

Reporting Summary

Nature Portfolio wishes to improve the reproducibility of the work that we publish. This form provides structure for consistency and transparency in reporting. For further information on Nature Portfolio policies, see our Editorial Policies and the Editorial Policy Checklist.

Statistics

For all statistical analyses, confirm that the following items are present in the figure legend, table legend, main text, or Methods section.

n/a	Confirmed	
☐	☒	The exact sample size (*n*) for each experimental group/condition, given as a discrete number and unit of measurement
☐	☒	A statement on whether measurements were taken from distinct samples or whether the same sample was measured repeatedly
☐	☒	The statistical test(s) used AND whether they are one- or two-sided<br>*Only common tests should be described solely by name; describe more complex techniques in the Methods section.*
☒	☐	A description of all covariates tested
☐	☒	A description of any assumptions or corrections, such as tests of normality and adjustment for multiple comparisons
☐	☒	A full description of the statistical parameters including central tendency (e.g. means) or other basic estimates (e.g. regression coefficient) AND variation (e.g. standard deviation) or associated estimates of uncertainty (e.g. confidence intervals)
☐	☒	For null hypothesis testing, the test statistic (e.g. *F*, *t*, *r*) with confidence intervals, effect sizes, degrees of freedom and *P* value noted<br>*Give P values as exact values whenever suitable.*
☒	☐	For Bayesian analysis, information on the choice of priors and Markov chain Monte Carlo settings
☒	☐	For hierarchical and complex designs, identification of the appropriate level for tests and full reporting of outcomes
☒	☐	Estimates of effect sizes (e.g. Cohen's *d*, Pearson's *r*), indicating how they were calculated

Our web collection on statistics for biologists contains articles on many of the points above.

Software and code

Policy information about availability of computer code

Data collection	Deltavision Ultra (GE Healthcare): AcquireUltra (version 1.2.3)<br>Abberior STEDYCON: STEDYCON smart control (version 7.1.53);<br>BD FACSCalibur: BD CellQuest Pro (4.0.2);<br>Zeiss Axio Imager A2: Zeiss ZEN Blue (3.3)<br>ChemiDoc MP Imaging System (Bio-Rad): Image Lab Software (2.4.0.03);<br>Amersham Typhoon phosphor imager (Cytiva): Amersham Typhoon control software (3.0.0.2)
Data analysis	Cytological and live-cell image analyses were performed using Fiji (version 2.14.0/1.54f). Live-cell and STED images were deconvolved with Huygens Professional (SVI, version 25.04) and analyzed using Fiji. Western blots were quantified in Fiji and prepared for presentation in Fiji and Adobe Photoshop (version 25.12.0). FACS data were analyzed using FlowJo (version 10.9.0). Southern blots were quantified with ImageQuant TL (version 8.1) or Fiji and adapted for presentation in Fiji. Graphs were generated, and all statistical analyses were performed in GraphPad Prism (version 9.5.1) or Microsoft Excel for Mac (version 16.87). Figures and schemes were assembled in Adobe Illustrator (version 25.0, 2021).

For manuscripts utilizing custom algorithms or software that are central to the research but not yet described in published literature, software must be made available to editors and reviewers. We strongly encourage code deposition in a community repository (e.g. GitHub). See the Nature Portfolio guidelines for submitting code & software for further information.

Data

Policy information about availability of data

All manuscripts must include a data availability statement. This statement should provide the following information, where applicable:

- Accession codes, unique identifiers, or web links for publicly available datasets
- A description of any restrictions on data availability
- For clinical datasets or third party data, please ensure that the statement adheres to our policy

Relevant data supporting the findings of this study are provided within the article and its supplementary Information. Source data for all images, Southern blots, and Western blots are available at DOI: 10.5281/zenodo.15862742. Biological materials used in this study are available from the corresponding author upon reasonable request.

Research involving human participants, their data, or biological material

Policy information about studies with human participants or human data. See also policy information about sex, gender (identity/presentation), and sexual orientation and race, ethnicity and racism.

Reporting on sex and gender	The study did not involve human participants, their data, or biological material.
Reporting on race, ethnicity, or other socially relevant groupings	The study did not involve human participants, their data, or biological material.
Population characteristics	The study did not involve human participants, their data, or biological material.
Recruitment	The study did not involve human participants, their data, or biological material.
Ethics oversight	The study did not involve human participants, their data, or biological material.

Note that full information on the approval of the study protocol must also be provided in the manuscript.

Field-specific reporting

Please select the one below that is the best fit for your research. If you are not sure, read the appropriate sections before making your selection.

☒ Life sciences ☐ Behavioural & social sciences ☐ Ecological, evolutionary & environmental sciences

For a reference copy of the document with all sections, see nature.com/documents/nr-reporting-summary-flat.pdf

Life sciences study design

All studies must disclose on these points even when the disclosure is negative.

Sample size	All sample sizes and the number of biological replicates for each experiment are reported in the figure legends. No statistical methods were used to predetermine sample size. For cytological and live-cell imaging analyses, sample sizes were guided by preliminary experiments that helped establish an appropriate sample size for the purpose of this study. For Southern and Western blot assays, the number of experiments performed followed standard practices in the field.
Data exclusions	No data were excluded from the analyses.
Replication	Experimental findings were confirmed by performing multiple biological replicates as indicated in the figure legends.
Randomization	Not applicable. Experiments involved comparisons between control (wild type or other appropriate controls) and mutant or treated yeast cells; randomization was therefore neither necessary nor appropriate.
Blinding	Blinding was not used during data collection and analysis. All results involved side-by-side comparisons of mutant or treated yeast cells with appropriate controls. For cytology experiments, blinding was not feasible because treated yeast cells typically exhibit distinct and specific features, such as disassembly of the synaptonemal complex.

Reporting for specific materials, systems and methods

We require information from authors about some types of materials, experimental systems and methods used in many studies. Here, indicate whether each material, system or method listed is relevant to your study. If you are not sure if a list item applies to your research, read the appropriate section before selecting a response.

Materials & experimental systems

n/a	Involved in the study	
☐	☒	Antibodies
☐	☒	Eukaryotic cell lines
☒	☐	Palaeontology and archaeology
☒	☐	Animals and other organisms
☒	☐	Clinical data
☒	☐	Dual use research of concern
☒	☐	Plants

Methods

n/a	Involved in the study	
☒	☐	ChIP-seq
☒	☐	Flow cytometry
☒	☐	MRI-based neuroimaging

Antibodies

Antibodies used

Primary antibodies for cytological analysis included: rabbit anti-Zip1 (Grigaitis et al., 2020), guinea pig anti-Rec8 (Bommi et al., 2019), rabbit anti-Zip3 (Shinohara et al., 2008), rabbit anti-Msh5 (Shinohara et al., 2008), mouse anti-Myc (9E10, Cancer Research UK), guinea pig anti-Ecm11-Gmc2 (Voelkel-Meiman et al., 2019), mouse anti-Smt3/SUMO (4F2.F5.G2, Rockland Immunochemicals), guinea pig anti-Hop1 (Iwasaki et al., 2016), mouse anti-γ-tubulin/Tub4 (MPI-CBG A81; Matos et al., 2008), and mouse anti-HA.11 (16B12, BioLegend).

Secondary antibodies for cytology were goat or donkey antibodies conjugated to Alexa Fluor 488, Alexa Fluor 555, and Alexa Fluor 647 (Invitrogen). For STED microscopy, secondary antibodies included goat anti-rabbit STAR ORANGE (Abberior) and goat anti-guinea pig STAR RED (Abberior).

Primary antibodies for Western blotting were: rabbit anti-Myc conjugated to HRP (ab1326, Abcam), rabbit anti-Zip1 (Grigaitis et al., 2020), rabbit anti-Crm1 (gift from K. Weis), mouse anti-Myc (9E10, Cancer Research UK; 1:5000), mouse anti-GFP (clones 7.1/13.1, Roche; 1:2000), rabbit anti-Ecm11 (gift from A.Pichler), rabbit anti-Smt3/SUMO (gift from A. Pichler), mouse anti-Pgk1 (22C5D8, Invitrogen), guinea pig anti-Hop1 (Iwasaki et al., 2016), rabbit anti-Hop1-pT318 (Iwasaki et al., 2016), and mouse anti-HA.11 (16B12, BioLegend).

Secondary antibodies for Western blotting included goat anti-mouse IgG conjugated to HRP (P0447, Agilent), swine anti-rabbit IgG conjugated to HRP (P0399, Agilent), goat anti-rabbit conjugated to IRDye 800CW (926-32211, LI-COR Biosciences), goat anti-mouse IgG conjugated to Alexa Fluor 680 (A21057, Invitrogen), and goat anti-guinea pig IgG conjugated to Alexa Fluor 647 (A21450, Invitrogen).

Validation

All home-made antibodies were characterized and validated with appropriate controls for both Western blotting and cytological analyses in previous publications, as cited above. For cytological and Western blot detection of tagged proteins using anti-Myc, anti-HA, or anti-GFP antibodies, untagged controls were included to confirm specificity. The specificity of anti-Smt3/SUMO (4F2.F5.G2, Rockland Immunochemicals) for immunofluorescence was confirmed by our deSUMOylation experiment (Figure 3 and Extended Data Figure 6). The specificity of anti-Crm1 (gift from K. Weis) and anti-Pgk1 (22C5D8, Invitrogen) was validated by detection of bands at the appropriate molecular weights in Western blotting. The specificity of anti-Ecm11 and anti-Smt3/SUMO (gifts from A. Pichler) was confirmed using an ecm11Δ knockout strain and deSUMOylation experiment (Figure 3 and Extended Data Figure 6), respectively.

Eukaryotic cell lines

Policy information about cell lines and Sex and Gender in Research

Cell line source(s)

We used budding yeast strains, all derivatives of SK1. Detailed genotypes are provided in Supplementary Table 1.

Authentication

n/a

Mycoplasma contamination

n/a

Commonly misidentified lines (See ICLAC register)

n/a

Plants

Seed stocks

The study did not involve plant seeds.

Novel plant genotypes

The study did not involve plants.

Authentication

The study did not involve plants.
