# Supplementary material for: Holliday junction–ZMM protein feedback enables meiotic crossover assurance
Source: Nature. 2025 Sep 24;647(8090):766–75. doi: 10.1038/s41586-025-09559-x (PMC12630000; doi:10.1038/s41586-025-09559-x)
Supplement: Supplementary file 3 — A list of yeast strains used in this study (Supplementary Table 1) and the spore viability data for newly generated yeast strains to evaluate the functional impact of protein tagging (Supplementary Table 2). [file 41586_2025_9559_MOESM3_ESM.docx]

## **Supplementary Table 1. Yeast strains used in this study.**

| **Strain** | **Genotype*** | **Figure** |
| --- | --- | --- |
| YML10358 | *KanMX4::P_GAL1_-YEN1^ON^-18myc::URA3 ura3::P_GPD1_-GAL4(848).ER::URA3 ndt80Δ::NatMX4* | 1, 2, 4, S1, S2, S4, S9 |
| YML10607 | *KanMX4::P_GAL1_-YEN1^ON-ND^-18myc::URA3 ura3::P_GPD1_-GAL4(848).ER::URA3 ndt80Δ::NatMX4* | 1, S1 |
| YML13412 | ***homozygous:*** *fpr1∆::NatMX4 RPL13A-2xFKBP12::TRP1 REC104-FRB-3HA::KanMX6 KanMX4::P_GAL1_-YEN1^ON^-18myc::URA3 his3::P_GPD1_-GAL4(848).ER::HIS3 nuc1∆::HygroB ndt80∆::HIS3* ***heterozygous:*** *HIS4::LEU2-(BAMHI;ori) his4X::LEU2-(NgoMIV,+ori)-URA3* | 1, S3 |
| YML14326 | *NUP84-3xmCherry::KanMX4 ZIP1::GFP^700^::HphMX4 KanMX4::P_GAL1_-YEN1^ON-ND^-18myc::URA3 ura3::P_GPD1_-GAL4(848).ER::URA3 ndt80Δ::NatMX4* | S1 |
| YML14327 | *NUP84-3xmCherry::KanMX4 ZIP1::GFP^700^::HphMX4 KanMX4::P_GAL1_-YEN1^ON^-18myc::URA3 ura3::P_GPD1_-GAL4(848).ER::URA3 ndt80Δ::NatMX4* | S1 |
| YML14994 | *ZIP4-9myc::KITRP1 ura3::P_GPD1_-GAL4(848).ER::URA3 trp1::P_GAL1_-YEN1^ON^-3FLAG-2TEV-10HIS::TRP1 ndt80Δ::NatMX4* | S4 |
| YML14881 | ***homozygous:*** *ZIP3-AID*-9myc::hphNT1 his3::P_CUP1_-osTIR1^F74G^::HIS3 ndt80Δ::NatMX4* ***heterozygous:*** *HIS4::LEU2-(BAMHI;ori) his4X::LEU2-(NgoMIV,+ori)-URA3* | 2, S4, S8 |
| YML14882 | ***homozygous:*** *ZIP3-AID*-9myc::hphNT1 SGS1-AID*-9myc::hphNT1 his3::P_CUP1_-osTIR1^F74G^::HIS3 ndt80Δ::NatMX4* ***heterozygous:*** *HIS4::LEU2-(BAMHI;ori) his4X::LEU2-(NgoMIV,+ori)-URA3* | 2 |
| YML15017 | *MSH4-AID*-9myc::hphNT1 his3::P_CUP1_-osTIR1^F74G^::HIS3 ndt80Δ::NatMX4* | 4, S4, S8 |
| YML15018 | *MSH4-AID*-9myc::hphNT1 SGS1-AID*-9myc::hphNT1 his3::P_CUP1_-osTIR1^F74G^::HIS3 ndt80Δ::NatMX4* | S5 |
| YML16509 | ***homozygous:*** *MSH4-AID*-9myc::hphNT1 his3::P_CUP1_-osTIR1^F74G^::HIS3 ndt80Δ::NatMX4*  ***heterozygous:*** *HIS4::LEU2-(BAMHI;ori) his4X::LEU2-(NgoMIV,+ori)-URA3* | S5 |
| YML16510 | ***homozygous:*** *MSH4-AID*-9myc::hphNT1 SGS1-AID*-9myc::hphNT1 his3::P_CUP1_-osTIR1^F74G^::HIS3 ndt80Δ::NatMX4*  ***heterozygous:*** *HIS4::LEU2-(BAMHI;ori) his4X::LEU2-(NgoMIV,+ori)-URA3* | S5 |
| YML14883 | ***homozygous:*** *SGS1-AID*-9myc::hphNT1 his3::P_CUP1_-osTIR1^F74G^::HIS3 ndt80Δ::NatMX4*  ***heterozygous:*** *HIS4::LEU2-(BAMHI;ori) his4X::LEU2-(NgoMIV,+ori)-URA3* | S5 |
| YML15019 | *ZIP4-AID*-9myc::hphNT1 his3::P_CUP1_-osTIR1^F74G^::HIS3 ndt80Δ::NatMX4* | S4, S8 |
| YML15020 | *ZIP4-AID*-9myc::hphNT1 SGS1-AID*-9myc::hphNT1 his3::P_CUP1_-osTIR1^F74G^::HIS3 ndt80Δ::NatMX4* | S5 |
| YML14908 | *NUP84-3xmCherry::KanMX4 ZIP1::GFP^700^::HphMX4 ZIP3-AID*-9myc::hphNT1 his3::P_CUP1_-osTIR1^F74G^::HIS3 ndt80Δ::NatMX4* | S5 |
| YML14909 | *NUP84-3xmCherry::KanMX4 ZIP1::GFP^700^::HphMX4 ZIP3-AID*-9myc::hphNT1 SGS1-AID*-9myc::hphNT1 his3::P_CUP1_-osTIR1^F74G^::HIS3 ndt80Δ::NatMX4* | S5 |
| YML15039 | ***homozygous:*** *REC104-AID*-9myc::hphNT1 fpr1∆::HygMX RPL13A-2xFKBP12::TRP1 his3::P_CUP1_-osTIR1^F74G^::HIS3 ura3::P_GPD1_-GAL4(848).ER::URA3 ndt80∆::HIS3* ***heterozygous:*** *natNT2::P_GAL1_-ulp1^∆(172-340)::GFP^-FRB::KanMX6* | 3, S6 |
| YML12424 | ***homozygous:*** *ura3::P_GPD1_-GAL4(848).ER::URA3 nuc1∆::HygroB ndt80Δ::NatMX4* ***heterozygous:*** *KanMX4::P_GAL1_-ulp1^∆(172-340)::GFP^ HIS4::LEU2-(BAMHI;ori) his4X::LEU2-(NgoMIV,+ori)-URA3* | 3, S6 |
| YML14134 | ***homozygous:*** *KanMX4::P_GAL1_-YEN1^ON^-18myc::URA3 fpr1∆::HygMX RPL13A-2xFKBP12::TRP1 ura3::P_GPD1_-GAL4(848).ER::URA3 ndt80Δ::HIS3* ***heterozygous:*** *natNT2::P_GAL1_-ulp1^∆(172-340)::GFP^-FRB::KanMX6* | 3, S7 |
| YML13542 | ***homozygous:*** *fpr1∆::HygMX RPL13A-2xFKBP12::TRP1 ura3::P_GPD1_-GAL4(848).ER::URA3 ndt80Δ::HIS3* ***heterozygous:*** *natNT2::P_GAL1_-ulp1^∆(172-340)::GFP^-FRB::KanMX6* | S7 |
| YML14887 | ***homozygous:*** *NUP84-3xmCherry::KanMX4 ZIP1::GFP^700^::HphMX4 fpr1∆::HygMX RPL13A-2xFKBP12::TRP1 ura3::P_GPD1_-GAL4(848).ER::URA3 ndt80Δ::HIS3* ***heterozygous:*** *natNT2::P_GAL1_-ulp1^∆(172-340)::GFP^-FRB::KanMX6* | 3, S7 |
| YML14273 | ***homozygous:*** *NUP84-3xmCherry::KanMX4 ZIP1::GFP^700^::HphMX4 KanMX4::P_GAL1_-YEN1^ON^-18myc::URA3 fpr1∆::HygMX RPL13A-2xFKBP12::TRP1 ura3::P_GPD1_-GAL4(848).ER::URA3 ndt80Δ::HIS3* ***heterozygous:*** *natNT2::P_GAL1_-ulp1^∆(172-340)::GFP^-FRB::KanMX6* | 3 |
| YML13542 | ***homozygous:*** *fpr1∆::HygMX RPL13A-2xFKBP12::TRP1 ura3::P_GPD1_-GAL4(848).ER::URA3 ndt80Δ::HIS3* ***heterozygous:*** *natNT2::P_GAL1_-ulp1^∆(172-340)::GFP^-FRB::KanMX6* | S7 |
| YML15784 | ***homozygous:*** *SAE2-AID*-9myc::hphNT1 his3::P_CUP1_-osTIR1^F74G^::HIS3 KanMX4::P_GAL1_-YEN1^ON^-18myc::URA3 ura3::P_GPD1_-GAL4(848).ER::URA3 ndt80Δ::NatMX4* ***heterozygous****: HIS4::LEU2-(BAMHI;ori) his4X::LEU2-(NgoMIV,+ori)-URA3* | 4, S8 |
| YML14918 | *NUP84-3xmCherry::KanMX4 ZIP1::GFP^700^::HphMX4 KanMX4::P_GAL1_-YEN1^ON^-18myc::URA3 ura3::P_GPD1_-GAL4(848).ER::URA3* | 4, S9 |
| YML16571 | *HTB1-mCherry::HIS3MX6 ZIP1::GFP^700^::HphMX4 KanMX4::P_CUP1_-YEN1^ON^-18myc::URA3* TRP1::P_GAL1_-NDT80 *ura3::P_GPD1_-GAL4(848).ER::URA3* | S9 |
| YML15435 | *ura3::P_GAL1_-CDC5-3HA::URA3 his3::P_GPD1_-GAL4(848).ER::HIS3 ndt80Δ::HIS3* | 5, S10 |
| YML15325 | *mlh3∆::KanMX6 P_CLB2_-3HA-MMS4::KanMX6 slx1∆::hphMX6 yen1∆::natMX4 ura3::P_GAL1_-CDC5-3HA::URA3 his3::P_GPD1_-GAL4(848).ER::HIS3 ndt80Δ::HIS3* | 5, S10 |
| YML16622 | *mlh3∆::KanMX6 P_CLB2_-3HA-MMS4::KanMX6 P_CLB2_-3HA-SGS1::KanMX6 slx1∆::hphMX6 yen1∆::natMX4 ura3::P_GAL1_-CDC5-3HA::URA3 his3::P_GPD1_-GAL4(848).ER::HIS3 ndt80Δ::HIS3* | S10 |
| YML13538 | *NUP84-3xmCherry::KanMX4 ZIP1::GFP^700^::HphMX4 ura3::P_GAL1_-CDC5-3HA::URA3 his3::P_GPD1_-GAL4(848).ER::HIS3 ndt80Δ::HIS3* | 5, S10 |
| YML14992 | *NUP84-3xmCherry::KanMX4 ZIP1::GFP^700^::HphMX4 mlh3∆::KanMX6 P_CLB2_-3HA-MMS4::KanMX6 slx1∆::hphMX6 yen1∆::natMX4 ura3::P_GAL1_-CDC5-3HA::URA3 his3::P_GPD1_-GAL4(848).ER::HIS3 ndt80Δ::HIS3* | 5 |
| YML16716 | *ZIP1::GFP^700^::HphMX4 mlh3∆::KanMX6 P_CLB2_-3HA-MMS4::KanMX6 P_CLB2_-3HA-SGS1::KanMX6 slx1∆::hphMX6 yen1∆::natMX4 ura3::P_GAL1_-CDC5-3HA::URA3 his3::P_GPD1_-GAL4(848).ER::HIS3 ndt80Δ::HIS3* | S10 |
| YML13523 | *ZIP1::GFP^700^::HphMX4 CNM67-tdTomato::NatMX4* | 5 |
| YML13498 | *ZIP1::GFP^700^::HphMX4 CNM67-tdTomato::NatMX4 mlh3∆::KanMX6 P_CLB2_-3HA-MMS4::KanMX6 slx1∆::hphMX6 yen1∆::natMX4* | 5 |
| YML16745 | *ZIP1::GFP^700^::HphMX4 CNM67-tdTomato::NatMX4 mlh3∆::KanMX6 P_CLB2_-3HA-MMS4::KanMX6 P_CLB2_-3HA-SGS1::KanMX6 slx1∆::hphMX6 yen1∆::natMX4* | 5 |
| YML5909 | *‘Wild type’* | S10 |
| YML12949 | *mlh3∆::KanMX6 P_CLB2_-3HA-MMS4::KanMX6 slx1∆::hphMX6 yen1∆::natMX4* | S10 |
| YML16744 | *mlh3∆::KanMX6 P_CLB2_-3HA-MMS4::KanMX6 P_CLB2_-3HA-SGS1::KanMX6 slx1∆::hphMX6 yen1∆::natMX4 ura3::P_GAL1_-CDC5-3HA::URA3 his3::P_GPD1_-GAL4(848).ER::HIS3* | S10 |

* All strains are diploid SK1 derivatives (*MATa/MATalpha ho::LYS2 or ho::hisG, his3::hisG leu2::hisG trp1::hisG ura3*) with genetic modifications at the endogenous locus unless stated otherwise.

## **Supplementary Table 2. Spore viability of newly generated strains to assess the functional impact of protein tagging.**

| **Strain** | **Genotype*** | **Dissected spores** | **Viable spores** | **Spore viability (%)** |
| --- | --- | --- | --- | --- |
| YML10774 | *wild type* | 216 | 215 | 99.5 |
| YML12992 | *ZIP3-AID*-9myc::hphNT* | 216 | 214 | 99.1 |
| YML4860 | *zip3∆::KanMX6* | 216 | 20 | 9.3 |
| YML14610 | *ZIP4-9myc::KITRP1* | 216 | 201 | 93.1 |
| YML14611 | *ZIP4-AID*-9myc::hphNT* | 216 | 205 | 94.9 |
| YML8588 | *zip4∆::KanMX6* | 216 | 55 | 25.5 |
| YML16581 | *MSH4-AID*-9myc::hphNT* | 216 | 216 | 100.0 |
| YML3972 | *msh4∆::KanMX6* | 216 | 62 | 28.7 |
| YML12792 | *SGS1-AID*-9myc::hphNT* | 216 | 212 | 98.1 |
| YML4668 | *sgs1∆::HIS3* | 216 | 160 | 74.1 |
| YML14922 | *REC104-FRB-3HA::KanMX6* | 216 | 213 | 98.6 |
| YML14921 | *REC104-AID*-9myc::hphNT* | 216 | 216 | 100.0 |
| YML16620 | *rec104∆::TRP1* | 216 | 5 | 2.3 |
| YML16579 | *SAE2-AID*-9myc::hphNT1* | 216 | 213 | 98.6 |
| YML16578 | *sae2∆::LEU2* | 216 | 2 | 0.9 |
| YML13675 | *NUP83-3xmCherry::KanMX4* | 216 | 215 | 99.5 |
| YML16701 | *nup84∆::TRP1* | 216 | 143 | 66.2 |

* All strains are diploid SK1 derivatives (*MATa/MATalpha ho::LYS2 or ho::hisG, his3::hisG leu2::hisG trp1::hisG ura3*) with genetic modifications at the endogenous locus unless stated otherwise.
