# Supplementary material for: Holliday junction–ZMM protein feedback enables meiotic crossover assurance
Source: Nature. 2025 Sep 24;647(8090):766–75. doi: 10.1038/s41586-025-09559-x (PMC12630000; doi:10.1038/s41586-025-09559-x)
Supplement: Supplementary file 4 — The legends to Supplementary Videos 1–17. [file 41586_2025_9559_MOESM4_ESM.docx]

**Supplementary Video 1.**

Time-lapse video of a pachytene-stage nucleus in a *P_GAL1_-YEN1^ON^ ndt80∆* cell expressing Zip1^GFP^, as shown in Extended Data Fig. 1h. β-estradiol was added at ~7h in SPM (t = 0 min) to induce Yen1^ON^ expression.

**Supplementary Video 2.**

Time-lapse video of a pachytene-stage nucleus in a *P_GAL1_-YEN1^ON-ND^ ndt80∆* cell expressing Zip1^GFP^, as shown in Extended Data Fig. 1j. β-estradiol was added at ~7h in SPM (t = 0 min) to induce Yen1^ON-ND^ expression.

**Supplementary Video 3.**

Time-lapse video of a pachytene-stage nucleus in a *ZIP3^AID^ ndt80∆* cell expressing Zip1^GFP^, as shown in Extended Data Fig. 5l (top panel). CuSO_4_ was added 6.5 hrs after meiotic induction in SPM to express osTir1^F74G^. 5-Ph-IAA was added at ~7h in SPM (t = 0 min) to deplete Zip3^AID^.

**Supplementary Video 4.**

Time-lapse video of a pachytene-stage nucleus in a *ZIP3^AID^ SGS1^AID^ ndt80∆* cell expressing Zip1^GFP^, as shown in Extended Data Fig. 5l (bottom panel). CuSO_4_ was added 6.5 hrs after meiotic induction in SPM to express osTir1^F74G^. 5-Ph-IAA was added at ~7h in SPM (t = 0 min) to deplete Zip3^AID^ and Sgs1^AID^.

**Supplementary Video 5.**

Time-lapse video of a pachytene-stage nucleus in a *P_GAL1_-ulp1^∆N-FRB^/ULP1 ndt80∆* cell expressing Zip1^GFP^, as shown in Extended Data Fig. 7d. β-estradiol was added at ~7h in SPM (t = 0 min) to induce Ulp1^∆N-FRB^ expression. DMSO was added after 120 min as a control.

**Supplementary Video 6.**

Time-lapse video of a pachytene-stage nucleus in a *P_GAL1_-ulp1^∆N-FRB^/ULP1 ndt80∆* cell expressing Zip1^GFP^, as shown in Fig. 3i. β-estradiol was added at ~7h in SPM (t = 0 min) to induce Ulp1^∆N-FRB^ expression. Rapamycin was added after 120 min to induce nuclear depletion of Ulp1^∆N-FRB^.

**Supplementary Video 7.**

Time-lapse video of a pachytene-stage nucleus in a *P_GAL1_-YEN1^ON^ P_GAL1_-ulp1^∆N-FRB^/ULP1 ndt80∆* cell expressing Zip1^GFP^, as shown in Fig. 3k. β-estradiol was added at ~7h in SPM (t = 0 min) to induce Ulp1^∆N-FRB^ and Yen1^ON^ expression. Rapamycin was added after 120 min to induce nuclear depletion of Ulp1^∆N-FRB^.

**Supplementary Video 8.**

Time-lapse video of a nucleus in a *P_GAL1_-YEN1^ON^* cell expressing Zip1^GFP^ and Nup84^mCherry^ undergoing meiosis and gametogenesis, as shown in Extended Data Fig. 9b. Time is relative to the first appearance of the structured Zip1^GFP^ signal (t = 0 min), indicating the onset of the zygotene stage.

**Supplementary Video 9.**

Time-lapse video of an early to mid zygotene-stage nucleus in a *P_GAL1_-YEN1^ON^* cell expressing Zip1^GFP^ and Nup84^mCherry^, as shown in Fig. 4i. Time is relative to the addition of β-estradiol (t = 0 min) to induce Yen1^ON^ expression.

**Supplementary Video 10.**

Time-lapse video of an early to mid zygotene-stage nucleus in a *P_GAL1_-YEN1^ON^* cell expressing Zip1^GFP^ and Nup84^mCherry^, as shown in Extended Data Fig. 9e. Time is relative to the addition of MeOH (t = 0 min) as a control.

**Supplementary Video 11.**

Time-lapse video of a pachytene-stage nucleus in a *P_GAL1_-YEN1^ON^* cell expressing Zip1^GFP^ and Nup84^mCherry^, corresponding to experiment in Extended Data Fig. 9g-i. Time is relative to the addition of β-estradiol (t = 0 min) to induce Yen1^ON^ expression.

**Supplementary Video 12.**

Time-lapse video of a pre-leptotene/leptotene-stage nucleus in a *P_GAL1_-YEN1^ON^* cell expressing Zip1^GFP^ and Nup84^mCherry^, corresponding to experiment in Extended Data Fig. 9j-l. Time is relative to the addition of β-estradiol (t = 0 min) to induce Yen1^ON^ expression.

**Supplementary Video 13.**

Time-lapse video of a pachytene-stage nucleus in a *P_CUP1_-YEN1^ON^ P_GAL1_-NDT80* cell expressing Zip1^GFP^ and Htb1^mCherry^, as shown in Fig. 9m. H_2_O was added at ~7h in SPM (t = -65 min) as a control, followed by β-estradiol addition at t = 0 min to induce Ndt80 expression.

**Supplementary Video 14.**

Time-lapse video of a pachytene-stage nucleus in a *P_CUP1_-YEN1^ON^ P_GAL1_-NDT80* cell expressing Zip1^GFP^ and Htb1^mCherry^, as shown in Fig. 9o. CuSO_4_ was added at ~7h in SPM (t = -65 min) to induce Yen1^ON^ expression, followed by β-estradiol addition at t = 0 min to induce Ndt80 expression.

**Supplementary Video 15.**

Time-lapse video of a pachytene-stage nucleus in a *P_GAL1_-CDC5 ndt80∆* cell expressing Zip1^GFP^, as shown in Fig. 5c (left panel). β-estradiol was added at ~7h in SPM (t = 0 min) to induce Cdc5 expression.

**Supplementary Video 16.**

Time-lapse video of a pachytene-stage nucleus in a *P_GAL1_-CDC5 mlh3∆ mms4^mn^ slx1∆ yen1∆ ndt80∆* cell expressing Zip1^GFP^, as shown in Fig. 5c (right panel). β-estradiol was added at ~7h in SPM (t = 0 min) to induce Cdc5 expression.

**Supplementary Video 17.**

Time-lapse video of a pachytene-stage nucleus in a *P_GAL1_-CDC5 mlh3∆ mms4^mn^ sgs1^mn^ slx1∆ yen1∆ ndt80∆* cell expressing Zip1^GFP^, as shown in Extended Data Fig. 10h (bottom panel). β-estradiol was added at ~7h in SPM (t = 0 min) to induce Cdc5 expression.
